# Comparison of ACUITY, CRUSADE, and GRACE Risk Scales for Predicting Clinical Outcomes in Patients Treated with Dual-Antiplatelet Therapy

**DOI:** 10.1055/s-0038-1675576

**Published:** 2018-11-27

**Authors:** Sun Young Choi, Moo Hyun Kim, Victor Serebruany

**Affiliations:** 1 Department of Biomedical Laboratory Science, Daegu Health College, Daegu, Republic of Korea; 2 Department of Cardiology, College of Medicine, Dong-A University, Busan, Republic of Korea; 3 HeartDrug™ Research Laboratories, Johns Hopkins University, Towson, Maryland, United States

**Keywords:** ACUITY, CRUSADE, GRACE, ACS, risk score, bleeding, dual-antiplatelet therapy

## Abstract

Several reliable scales have been proposed for the management and prognosis in patients with acute coronary syndromes (ACS) treated with dual-antiplatelet therapy (DAPT). We sought to compare the performance of three conventional risk scores to predict major bleeding (MB; such as ACUITY or CRUSADE), or major adverse cardiovascular event (MACE for GRACE). This study included 904 consecutive post-ACS patients from the single Korean study center who underwent coronary interventions, and were treated with DAPT. All three scores were calculated based on admission data. MB and MACE were collected at 30-day and 1-year follow-ups. MB was defined according to the Bleeding Academic Research Consortium (BARC) criteria (types 3–5), and MACE included all-cause death, myocardial infarction, target vessel revascularization, and stroke. MB occurred in 114 patients (12.6%) during 30 days, and 65 patients (7.2%) from 30 days till 1-year follow-up. MACE occurred in 28 (3.1%) and 72 (8.0%) patients during 30 and 30 days till 1 year, respectively. For 30 days MB, the discriminatory ability of ACUITY (AUC: 0.83, 95% CI: 0.81–0.86) and CRUSADE (AUC: 0.82, 95% CI: 0.79–0.84) was similar, and more reliable than GRACE (AUC: 0.74, 95% CI: 0.71–0.77; *p* < 0.0001 and *p* = 0.002, respectively). The predictive value for 1-year MB was similar between ACUITY (AUC: 0.75, 95% CI: 0.72–0.78, *p* < 0.0001), CRUSADE (AUC: 0.70, 95% CI: 0.70–0.73, *p* < 0.0001), and GRACE (AUC: 0.70, 95% CI: 0.67–0.73, *p* < 0.0001) classifications. All three risk scales exhibited similar prediction for 30-day and 1-year MACE. We conclude that ACUITY and CRUSADE scores were superior to GRACE in predicting 30-day MB. However, all three risk scales were similarly useful for long-term MB, and MACE assessment.

## Introduction

Dual-antiplatelet therapy (DAPT) with aspirin and a P2Y _12_ inhibitor after coronary intervention with stent implantation reduces ischemic events but increase bleeding risk, which has been associated with a critical adverse event. 1 2 Numerous risk assessment scales have been introduced to better identify high-risk patients prone to bleeding and ischemic risk 3 4 5 6 7 or vice versa. The development of a simple-to-use risk scores for bleeding and ischemia could standardize therapeutic decision making and clinical outcomes. ACUITY (Acute Catheterization and Urgent Intervention Triage Strategy) 3 and CRUSADE (Can Rapid Risk Stratification of Unstable Angina Patients Suppress Adverse Outcomes with Early Implementation of the ACC/AHA Guidelines) 4 scores are bleeding risk algorithms. In contrast, GRACE (The Global Registry of Acute Coronary Events) 5 score targeted future ischemic events and mortality. Most ischemic and bleeding risk algorithms were derived from clinical trials, and include conventional clinical and laboratory characteristics, predominantly focusing on short-term events. There are numerous reports comparing predictive values of different scales and conventional biomarkers, with somewhat mixed results. 6 7 We recently show that CRUSADE score was superior to platelet testing for predicting short-term, but not 1-year, bleeding in post–percutaneous coronary intervention (PCI) Korean patients treated with DAPT. 8

The aim of this study was to compare the predictive performance of CRUSADE, ACUITY, and GRACE risk scores for short-term and long-term thrombotic and bleeding events in patients treated with DAPT.

## Methods

### Patients

A total of 904 consecutive post-PCI patients were included (Dong-A University Medical Center, Busan, Korea). All patients received maintenance DAPT (75 mg/day clopidogrel, or 10 mg/day prasugrel, or 180 mg/day ticagrelor, all on top of 100 mg aspirin), and were included in the prospective observational cross-sectional study. Written informed consent was obtained from all patients, and the study protocol was approved by the Ethical Review Board of Dong-A University Hospital. Exclusion criteria were DAPT maintenance <1 year, hemodynamic instability, malignancies, active bleeding or major surgery within 4 weeks, severe chronic renal failure, and treatment with other types of antiplatelet agents (e.g., cilostazol, or glycoprotein IIb/IIIa receptor blocker).

### Outcomes

Major bleeding (MB) was defined according to the Bleeding Academic Research Consortium (BARC) criteria (type 3 or 5: hemodynamic instability, need for transfusion, drop in hemoglobin ≥ 3 g, and intracranial, intraocular, or fatal bleeding). 9 Major adverse cardiovascular events (MACEs) were defined as all-cause death, myocardial infarction, target vessel revascularization, and stroke. Both MB and MACE were evaluated within 1 month, and then at 12 months of follow-up. The ACUITY, 3 CRUSADE, 4 and GRACE 5 risk scores were calculated from patients' clinical characteristics available in the hospital records. ACUITY score consists of seven variables (female sex, age, type of acute coronary syndrome [ACS], unstable angina, non-ST elevation or ST elevation acute myocardial infarction, serum creatinine, and white blood cell count, all analyzed as ordinal categories). CRUSADE score was assessed with the online calculator by eight variables (female sex, diabetes mellitus, chronic heart failure, valvular heart disease, heart rate, systolic blood pressure, glomerular filtration rate, and hematocrit). GRACE score was also examined with online calculator by eight variables (age, heart rate, systolic blood pressure, serum creatinine, Killip class, cardiac arrest at admission, elevated cardiac marker, ST segment deviation). The individual rating for each variable established in each score was assigned. The total score of each patient was calculated by summing the individual result for each prognostic variable included in the score. The patients were mandatorily contacted by telephone call, or underwent personal hospital visit for outcome assessment.

### Statistics

Continuous variables are expressed as means and standard deviations, while categorical variables were presented as frequencies (percentages). The comparisons between two mean values of continuous variables were analyzed using Student's *t* -test. Categorical variables were compared by using Pearson's chi-square or Fisher's exact test. The ACUITY, CRUSADE, and GRACE scores were stratified in three risk categories of bleeding as low, moderate, and high. 3 4 5 The predictive values of ACUITY, CRUSADE, and GRACE scores were assessed by receiver operating characteristics (ROC) curve analysis (using MedCalc Version 12.2.1; MedCalc Software, Mariakerke, Belgium), applying net reclassification and integrated discrimination improvement (IDI). 10 Prognostic utility of the risk models for MB and MACE has been assessed by deriving their C-statistics, using ROC curves. In general, a model with a C-statistic above 0.70 has acceptable discriminatory capacity. 11 The C-statistics for the three risk models were compared with each other using a nonparametric test. 12 Net reclassification improvement (NRI) represents the average weighted improvement in discrimination. IDI considers the change in the estimated prediction probabilities as a continuous variable and represents the average improvement in predicted probability. The impact of the reclassification procedure by using the superior score was assessed by using the method of NRI. Positive values of NRI indicate a predominance of correct reclassification, while negative values indicate a predominance of incorrect reclassification. A *p* -value < 0.05 rejects the null hypothesis of NRI = 0. 12 A *p* –value < 0.05 was considered to indicate significance. Statistical analyses were performed using SPSS version 18.0 (SPSS Inc., Chicago, Illinois, United States).

## Results

### Baseline Characteristics

The study cohort was composed of 904 patients treated with DAPT. The baseline demographics and clinical characteristics are exhibited in Table 1 . The MB occurred in 114 patients (12.6%) during the first 30 days, and extra 65 patients (7.2%) at 30 days to 1-year of follow-up. The MACE occurred in 28 (3.1%) and 72 (8.0%) patents during 30 and 30 days to 1 year, respectively. Background clinical variables and admission biomarkers were distributed differently, and depended on future MB and MACE outcomes. The future MB patients more commonly presented with non-ST-segment elevated myocardial infarction (NSTEMI) and ST-segment elevated myocardial infarction (STEMI), were older, and more frequent were females when compared with no MB patients. The MB was also associated with diabetes mellitus, hypertension, current smoking, previous stroke, and renal dysfunction. Similarly, MACE patients had a higher prevalence of elderly, diabetes, previous stroke, or MI, revascularization, and renal dysfunction than patients with no MACE. The mean ACUITY, CRUSADE, and GRACE scores were overall higher in patients who experienced MB and MACE compared with those without MB and MACE.

**Table 1:** Baseline characteristics in 904 ACS patients

Variables	MB ( *n* = 154)	No MB ( *n* = 750)	*p* -Value	MACE ( *n* = 97)	No MACE ( *n* = 807)	*p* -Value
Age, y	71.4 ± 9.4	64.1 ± 10.3	0.000	68.4 ± 10.1	65.1 ± 10.5	0.004
Female	74 (48.1)	197 (26.3)	0.000	32 (33.0)	239 (29.6)	0.282
BMI, kg/m ^2^	23.5 ± 3.3	24.6 ± 3.1	0.000	23.9 ± 2.9	24.5 ± 3.2	0.107
Diagnosis			0.000			0.083
Unstable angina	70 (45.5)	523 (69.7)		53 (54.6)	540 (66.9)	
NSTEMI	68 (44.2)	193 (25.7)		37 (38.1)	224 (27.8)	
STEMI	16 (10.4)	34 (4.5)		7 (7.2)	43 (5.3)	
Prior antiplatelet therapy	78 (50.6)	394 (52.5)	0.368	71 (73.2)	401 (49.7)	0.000
Risk factor						
Diabetes mellitus	77 (50.0)	307 (40.9)	0.024	51 (52.6)	333 (41.3)	0.022
Hypertension	123 (79.9)	472 (62.9)	0.000	74 (76.3)	521 (64.6)	0.013
Dyslipidemia	83 (53.9)	433 (57.7)	0.215	54 (55.7)	462 (57.2)	0.424
Current smoking	30 (19.5)	210 (28.0)	0.017	27 (27.8)	213 (26.4)	0.422
Past history						
Prior MI	36 (23.4)	183 (24.4)	0.438	36 (37.1)	183 (22.7)	0.002
Prior PCI	58 (37.7)	311 (41.5)	0.217	59 (60.8)	310 (38.4)	0.000
Prior stroke	27 (17.5)	66 (8.8)	0.002	18 (18.6)	75 (9.3)	0.006
Heart rate, bpm	83.5 ± 19.5	73.4 ± 13.8	0.000	78.8 ± 18.4	74.7 ± 15.0	0.012
Systolic BP, mm Hg	131.9 ± 27.1	129.7 ± 21.7	0.268	129.6 ± 21.9	130.1 ± 22.9	0.821
LVEF	55.5 ± 12.5	59.7 ± 10.5	0.000	55.2 ± 13.8	59.4 ± 10.6	0.000
Total cholesterol, mg/dL	166.5 ± 48.7	163.3 ± 39.1	0.404	152.7 ± 42.3	165.1 ± 40.4	0.007
WBC count (10 ^6^ /mL)	8.8 ± 3.8	7.8 ± 2.8	0.000	8.2 ± 3.0	7.9 ± 3.0	0.287
Platelets count, 10 ^3^ µL	214.3 ± 78.4	210.6 ± 58.2	0.498	204.0 ± 60.6	212.1 ± 62.2	0.226
Hemoglobin, g/dL	11.2 ± 2.1	13.1 ± 1.7	0.000	12.1 ± 2.3	12.9 ± 1.9	0.000
eGFR, mL/min, 1.73/m ^2^	60.9 ± 30.0	79.2 ± 24.1	0.000	60.3 ± 28.6	78.0 ± 25.1	0.000
Bleeding risk scores						
CRUSADE	43.7 ± 14.7	28.4 ± 12.6	0.000	39.8 ± 15.7	29.9 ± 13.7	0.000
GRACE	153.3 ± 45.4	118.3 ± 32.7	0.000	144.6 ± 44.2	121.8 ± 35.9	0.000
ACUITY	18.7 ± 6.3	11.0 ± 6.1	0.000	16.0 ± 7.3	11.8 ± 6.6	0.000

Abbreviations: BMI, body mass index; BP, blood pressure; eGFR, estimated glomerular filtration rate; MACE, major adverse cardiovascular event; MB, major bleeding; MI, myocardial infarction; LVEF, left ventricular ejection fraction; NSTEMI, non-ST-segment elevated myocardial infarction; PCI, percutaneous coronary intervention; STEMI, ST-segment elevated myocardial infarction.

### Outcomes

**Fig. 1:**
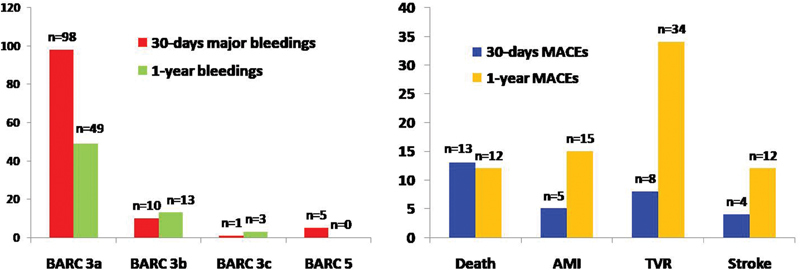
Distribution of major bleeding events by BARC scale and MACE.

**Fig. 2:**
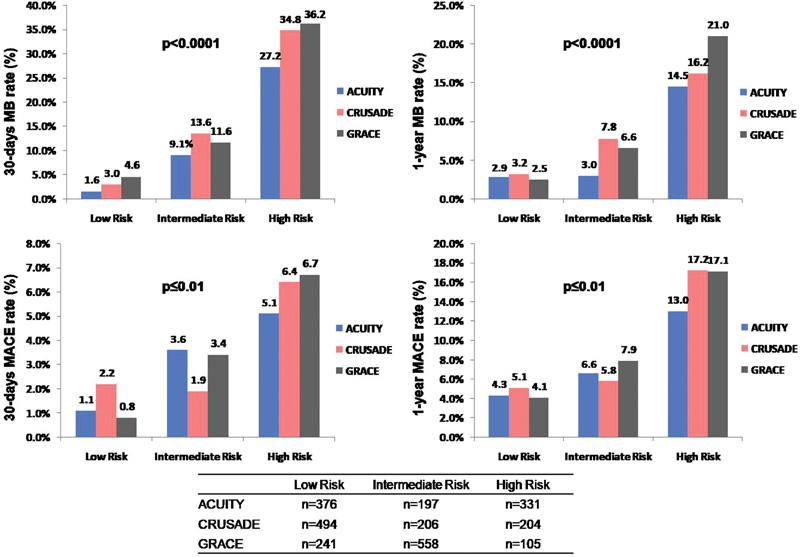
Incidence of major bleeding events and MACE across ACUITY, CRUSADE, and GRACE risk score categories.

The incidence of MB and MACE is presented in Fig. 1 . The BARC type 3a defined as blood transfusion or drop in hemoglobin ≥3 g/dL was observed in 98 and later in 49 patients at 30- and 30-day to 1-year follow-up, respectively. Among MACE, the most frequent outcomes were all-cause death with 13 cases for 30 days and target vessel revascularization with 34 cases for delayed 30-day to 1-year follow-up. The frequency of MB and MACE across ACUITY, CRUSADE, and GRACE risk scores is presented in Fig. 2 . By applying previously validated criteria, 331 patients (36.6%) based on ACUITY, 204 patients (22.6%) based on CRUSADE, and 105 patients (11.6%) based on GRACE scales met the threshold for the high bleeding risk category. The transition from a lower to a higher risk category carried a significant increase in MB and MACE rates across all risk scores.

### Predictive Value of Risks Scales

**Fig. 3:**
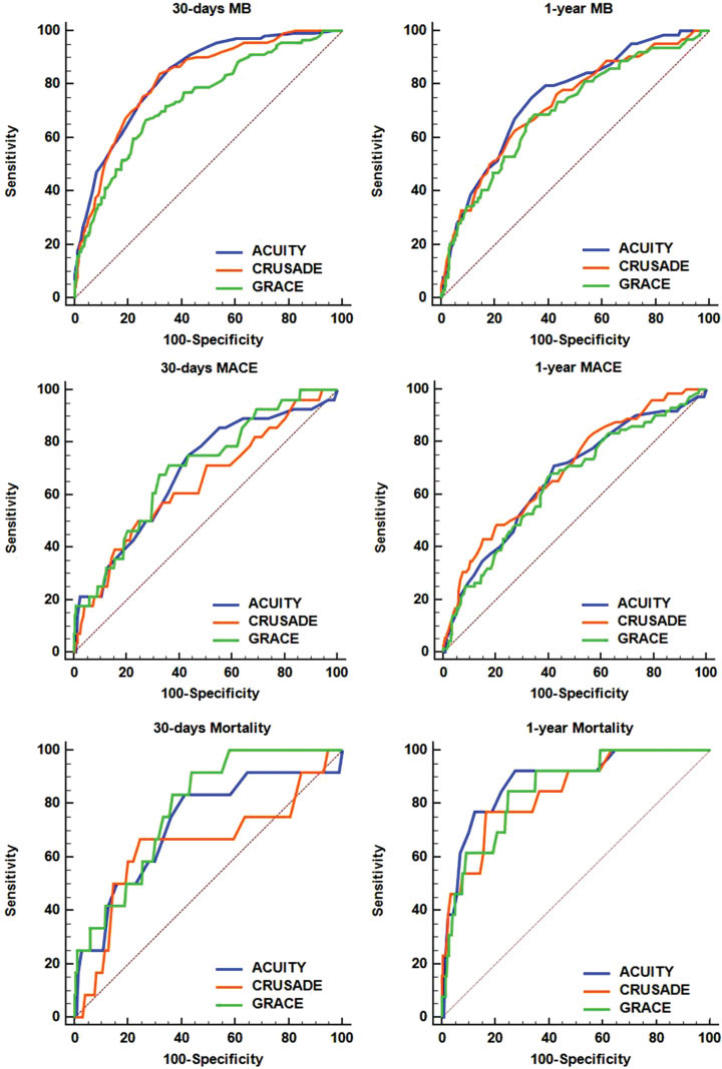
Receiver operating characteristic curves for predicting major bleeding and death at 30 days and 1 year.

Table 2 and Fig. 3 present the discriminatory capacity of three risk scores for MB and MACE, including assessing of the area under the curve (AUC). Applying C statistics, the discriminatory ability of ACUITY (AUC: 0.82, 95% CI: 0.80–0.85, *p* < 0.0001) and CRUSADE (AUC: 0.82, 95% CI: 0.79–0.84, *p* < 0.0001) for 30-day MB was similar ( *p* = 0.76), but better than GRACE (AUC: 0.74, 95% CI: 0.71–0.77, *p* < 0.0001) ( *p* < 0.001 and *p* = 0.01, respectively). These data are outlined in Table 3 . The point estimate of AUC for the prediction of 1-year MB was similar between ACUITY (AUC: 0.75, 95% CI: 0.72–0.77, *p* < 0.0001), CRUSADE (AUC: 0.72, 95% CI: 0.69–0.75, *p* < 0.0001), and GRACE (AUC: 0.70, 95% CI: 0.67–0.73, *p* < 0.0001). The point estimate of AUC for the prediction of the ACUITY, CRUSADE, and GRACE risk scores for 30-day and 1-year MACE, including death, was also not significantly different. ACUITY and CRUSADE risk scores successfully reclassified the risk of 30-day MB compared with GRACE risk scores ( Table 4 ). Importantly, the CRUSADE risk score was significantly superior to GRACE or ACUITY for reclassification improvement and IDI for 1-year MACE.

**Table 2:** Predictive performance of risk scores for major outcomes

Variables	30-d major bleeding		1-y major bleeding	
	C statistics (95% CI)	*p*	C statistics (95% CI)	*p*
ACUITY score	0.83 (0.81–0.86)	<0.0001	0.75 (0.72–0.78)	<0.0001
CRUSADE score	0.82 (0.79–0.84)	<0.0001	0.73 (0.70–0.76)	<0.0001
GRACE score	0.74 (0.71–0.77)	<0.0001	0.70 (0.67–0.73)	<0.0001
**Variables**	**30-d MACE**		**1-y MACE**	
	**C statistics (95% CI)**	***p***	**C statistics (95% CI)**	***p***
ACUITY score	0.69 (0.65–0.72)	0.0003	0.66 (0.63–0.70)	<0.0001
CRUSADE score	0.64 (0.61–0.67)	0.0101	0.69 (0.66–0.72)	<0.0001
GRACE score	0.70 (0.67–0.73)	0.0001	0.65 (0.61–0.68)	<0.0001
**Variables**	**30-d death**		**1-y death**	
	**C statistics (95% CI)**	***p***	**C statistics (95% CI)**	***p***
ACUITY score	0.72 (0.69–0.75)	0.006	0.88 (0.86–0.91)	<0.0001
CRUSADE score	0.64 (0.61–0.68)	0.127	0.84 (0.82–0.87)	<0.0001
GRACE score	0.78 (0.75–0.81)	<0.0001	0.85 (0.83–0.87)	<0.0001

Abbreviations: CI, confidence interval; MACE, major adverse cardiovascular event.

**Table 3:** Discrimination of ACUITY versus CRUSADE versus GRACE for predicting outcomes

Variables	30-d major bleeding		1-y major bleeding	
	z statistics	*p*	z statistics	*p*
ACUITY vs. CRUSADE	0.635	0.764	0.975	0.330
ACUITY vs. GRACE	4.222	<0.0001	1.624	0.104
CRUSADE vs. GRACE	3.117	0.002	0.707	0.450
**Variables**	**30-days MACE**		**1-year MACE**	
	**z statistics**	***p***	**z statistics**	***p***
ACUITY vs. CRUSADE	0.895	0.371	1.030	0.303
ACUITY vs. GRACE	0.279	0.780	0.556	0.578
CRUSADE vs. GRACE	1.012	0.311	1.329	0.184
**Variables**	**30-d death**		**1-y death**	
	**z statistics**	***p***	**z statistics**	***p***
ACUITY vs. CRUSADE	1.107	0.268	0.819	0.413
ACUITY vs. GRACE	0.978	0.328	0.643	0.520
CRUSADE vs. GRACE	1.670	0.095	0.148	0.882

Abbreviation: MACE, major adverse cardiovascular event.

**Table 4:** Risk reclassification and integrated discriminatory improvement for outcomes

Comparison	Event	Events correctly reclassified, P (n1)	Non-events correctly reclassified, P (n2)	NRI (95% CI)	*p*	IDI (95% CI)	*p*
CRUSADE vs. ACUITY a	30-d MB	0.39 (44)	0.39 (312)	−0.01 (−0.19∼0.17)	0.929	0.02 (0∼0.05)	0.070
	1-y MB	0.41 (26)	0.39 (330)	0.01 (−0.22∼0.25)	0.918	−0.01 (−0.02∼0)	0.259
	30-d MACE	0.21 (6)	0.26 (228)	−0.05 (−0.41∼0.32)	0.811	0 (−0.01∼0.01)	0.900
	1-y MACE	0.39 (28)	0.25 (206)	0.14 (−0.08∼0.36)	0.250	0.01 (0∼0.02)	0.031
	30-d death	0.5 (6)	0.37 (328)	0.13 (−0.36∼0.63)	0.649	0 (−0.01∼0.01)	0.736
	1-y death	0.69 (9)	0.63 (561)	0.06 (−0.33∼0.46)	0.822	0.01 (0∼0.01)	0.273
ACUITY vs. GRACE a	30-d MB	0.11 (12)	0.43 (342)	−0.54 (−0.73∼ − 0.35)	0.000	−0.05 (v0.07∼ − 0.02)	0.000
	1-y MB	0.03 (2)	0.07 (62)	−0.11 (−0.36∼0.15)	0.418	0.01 (−0.01∼0.02)	0.548
	30-d MACE	0.29 (8)	0.38 (330)	−0.09 (−0.45∼0.27)	0.636	0 (0∼0.01)	0.684
	1-y MACE	0.03 (2)	0.07 (58)	−0.04 (−0.28∼0.2)	0.733	0 (−0.01∼0.01)	0.695
	30-d death	0.17 (2)	0.07 (62)	0.24 (−0.33∼0.8)	0.416	0 (−0.01∼0.01)	0.871
	1-y death	0.23 (3)	0.07 (63)	0.3 (−0.23∼0.83)	0.281	0.02 (0∼0.04)	0.103
CRUSADE vs. GRACE a	30-d MB	0.14 (16)	0.37 (290)	−0.51 (−0.7∼ − 0.31)	0.000	−0.07 (−0.1∼ − 0.04)	0.000
	1-y MB	0 (0)	0.15 (122)	−0.15 (−0.4∼0.11)	0.263	0 (−0.02∼0.02)	0.933
	30-d MACE	0.35 (10)	0.19 (164)	0.17 (−0.18∼0.52)	0.376	0 (−0.01∼0.01)	0.838
	1-y MACE	0.22 (16)	0.6 (48)	−0.28 (−0.52∼ − 0.04)	0.023	−0.02 (−0.03∼0)	0.034
	30-d death	0.33 (4)	0.19 (170)	0.14 (−0.39∼0.68)	0.623	0 (−0.01∼0.01)	0.848
	1-y death	0.54 (7)	0.19 (167)	0.35 (−0.11∼0.81)	0.209	0.01 (−0.01∼0.03)	0.206

Abbreviations: MACE, major adverse cardiovascular event; MB, major bleeding.

a The model considered each bleeding risk score as a reference value for the others.

## Discussion

The main finding of this study is that among three conventional scores, ACUITY and CRUSADE risk scores demonstrated reasonably good predictive values with respect to short-term MB during DAPT when compared with GRACE. Moreover, originally designed as bleeding risk scores, they also displayed a similar capability to predict short-term and long-term mortality risks when compared with GRACE score. Importantly, the CRUSADE risk score predicted long-term MACE better than GRACE, when advanced statistics were applied. However, these data on comprehending CRUSADE and ACUITY success are somewhat mixed for two main reasons. First, patients differ substantially, somewhat neglecting that this useful score was designed exclusively for non-STEMI cohorts predicting very early in-hospital MB. Second, there are over dozen current bleeding classifications, and their inventors or/and promoters may be biased in applying their own scales at the expense of other useful algorithms. 13 14 Some other integrative models, such as HASBLED, are much more simple than CRUSADE, but it is unclear how they may be implemented for the similar delayed approach to pick up either bleeding or adverse thrombotic event signal. 15 Expanding original CRUSADE applicability beyond exclusive non-STEMI patients 4 to the entire post-ACS pool is also important, especially considering our data in Korean patients and those facts yielded from Egyptian study yielded similar results. 16 Finally, some evidence indicates the special difficulties in delayed bleeding prediction, 17 what is matching well with the index dataset. That message is particularly critical since late catastrophic hemorrhages are usually the most deadly, unexpected, and hard to prevent. Our data are in full agreement with another recent study suggesting that both CRUSADE and ACUITY risk scores performed adequate discriminatory power for the prediction of MB within 30 days in ticagrelor-treated ACS patients. 18 In fact, these three scores differ very significantly in our patient mixed cohort. ACUITY and GRACE include the wide scale of ACS, whereas CRUSADE applied almost exclusively to unstable angina. Furthermore, GRACE, being indicative of MACE rather than bleeding, is relatively easy to calculate and has the rather well-defined and accepted cut-off of 140 for high- (invasive) versus low-risk (conservative) patients. This consideration somewhat lowers the priority of this analyses to for clinicians. The unique meta-analyses pooling the evidence with regard to all three scales discussed here indicate their similar predictive value for bleeding risks, while accuracy of the scores increased with radial access for coronary interventions. 19

## Strengths and Limitations

Large sample size, reasonably validated and uniformed three established scales, and both short- and long-term observations with very careful follow-up are obvious assets. Single busy clinical center environment also reduces variability of techniques and outcomes. Each adverse event was clinically verified and confirmed. We applied the universal BARC bleeding classification, which was introduced to fairly and objectively count hemorrhages. This scale exhibits consistent growing popularity, and more frequently being validated in the major DAPT trials. Finally, we tested the abilities of three major risk scores simultaneously to discriminate outcomes, which was never done before. There are certain limitations worth mentioning. Importantly, applying a bleeding risk score to predict ischemic events (and vice versa) remains controversial. The inferior performance due to misuse of the scores is therefore not surprising. As the pathophysiology and predictors for bleeding and for MACE may differ, future studies should separately assess the comparative performance among ischemic risk scores (e.g., GRACE vs. TIMI vs. PURSUIT) and bleeding risk scores (e.g., CRUSADE vs. ACUITY). It should be emphasized that there might be important confounders to our analysis potentially impacting the conclusions including potentially missed outcomes. Among most important limitations are nonrandomized observational cross-sectional design, background differences among the patients, and pooled analyses of various stenting techniques (the index dataset). There were very few (∼10%) classical STEMI patients, limiting extrapolation of the index data to more “heavy” ischemic cohorts. We used a “real-life” registry, acknowledging that minority of prasugrel and ticagrelor patients may compromise the homogeneity of clopidogrel data, potentially increasing the statistical “noise.” Another shortcoming is the fact that we did not capture minor bleeding events, limiting the clinical applicability of the index dataset. Future studies should definitely focus more on minor hemorrhagic complications, which are critical for compliance, and drug discontinuations. In this study, we deliberately focus on delayed bleeding risks, also acknowledging that most bleeds occur early, and those were missed since we used up to 12 months' lag in capturing events. From a pragmatic point of view, considering recently discovered association of malignancies, potency of antiplatelet agents, and bleeding, 20 we now feel that it was a mistake to exclude cancer patients from this registry. It would be also useful to count minor bleeding events, which has not been done here. With respect to DAPT and especially for bleeding risk prediction, it would be interesting in the future to match how the real “DAPT risk scores” perform here. It will be important to include PRECISE-DAPT, DAPT, TIMI score, and/or PARIS score to the further analysis. 21 The first two specifically could be important as they are included in the recent ESC DAPT 2017 guidelines. Also, as with any conventional antiplatelet studies, with very few exceptions, compliance with drugs was not assessed. Finally, our study was conducted exclusively in Korean cohort; so, extrapolation of these facts into other ethnicities seems premature.

In conclusion, ACUITY and CRUSADE scores were superior to GRACE in predicting 30-day bleeding. However, all three risk scales were similarly reliable for long-term 1-year hemorrhages, and thrombotic events assessment in Korean patients. Further evidence should be urgently retrieved from large unbiased uniformed government national registries or large well-controlled insurance claims datasets.
