# Erratum to: “Comparison of ACUITY, CRUSADE, and GRACE Risk Scales for Predicting Clinical Outcomes in Patients Treated with Dual-Antiplatelet Therapy”

**DOI:** 10.1055/s-0039-1677685

**Published:** 2019-01-30

**Authors:** Sun Young Choi, Moo Hyun Kim, Victor Serebruany

**Affiliations:** 1 Department of Biomedical Laboratory Science, Daegu Health College, Daegu, Republic of Korea; 2 Department of Cardiology, College of Medicine, Dong-A University, Busan, Republic of Korea; 3 HeartDrug™ Research Laboratories, Johns Hopkins University, Towson, Maryland, United States

In the Original Article by Choi et al. “Comparison of ACUITY, CRUSADE, and GRACE Risk Scales for Predicting Clinical Outcomes in Patients Treated with Dual-Antiplatelet Therapy” ( TH Open 2018; 02(04): e399-e406 ) it has come to the authors attention that the wrong corresponding author was listed. The amended corresponding author address is:

Address for correspondence

Prof. Moo Hyun Kim

Department of Cardiology

College of Medicine

Dong-A University

Busan

Republic of Korea

(e-mail: KimMH@dau.ac.kr )

